# A computerized volumetric segmentation method applicable to multi-centre MRI data to support computer-aided breast tissue analysis, density assessment and lesion localization

**DOI:** 10.1007/s11517-016-1484-y

**Published:** 2016-04-22

**Authors:** Gokhan Ertas, Simon J. Doran, Martin O. Leach

**Affiliations:** 1 Cancer Research UK Cancer Imaging Centre, Division of Radiotherapy and Imaging, The Institute of Cancer Research, 123 Old Brompton Road, London, SW7 3RP UK; 2 Department of Biomedical Engineering, Yeditepe University, Istanbul, Turkey

**Keywords:** MRI, Breast, Segmentation, Fuzzy c-means, Multi-centre, Multi-instrument

## Abstract

Density assessment and lesion localization in breast MRI require accurate segmentation of breast tissues. A fast, computerized algorithm for volumetric breast segmentation, suitable for multi-centre data, has been developed, employing 3D bias-corrected fuzzy c-means clustering and morphological operations. The full breast extent is determined on T1-weighted images without prior information concerning breast anatomy. Left and right breasts are identified separately using automatic detection of the midsternum. Statistical analysis of breast volumes from eighty-two women scanned in a UK multi-centre study of MRI screening shows that the segmentation algorithm performs well when compared with manually corrected segmentation, with high relative overlap (RO), high true-positive volume fraction (TPVF) and low false-positive volume fraction (FPVF), and has an overall performance of RO 0.94 ± 0.05, TPVF 0.97 ± 0.03 and FPVF 0.04 ± 0.06, respectively (training: 0.93 ± 0.05, 0.97 ± 0.03 and 0.04 ± 0.06; test: 0.94 ± 0.05, 0.98 ± 0.02 and 0.05 ± 0.07).

## Introduction

Magnetic resonance imaging (MRI) is gaining increased acceptance for the diagnosis of breast cancer and the differential diagnosis of enhancing lesions [24], with enhancement information that reflects vascularity and permeability of breast tissues. When compared to X-ray mammography for screening women with a family history of breast cancer, breast MRI provides superior detection and classification of invasive cancer [23, 25, 26, 44]. Moreover, breast MRI offers the potential for accurate measurement of fibroglandular tissue volume to assess breast density, which is a strong risk factor associated with the development of breast cancer [44, 3, 19, 38]. To improve interpretation, accuracy and reproducibility further, there is considerable potential for computer assistance to process the large volume of image data produced during an MR investigation, which often has high spatial and temporal resolution [2].

For breast density assessment or lesion localization, computer-assisted MR image evaluation requires accurate separation of breast volume from other tissues and regions of the body. The breast–air boundary is identified easily by searching for a sharp increase in the image intensity from the air side provided that the background noise is low [9, 13, 22]. However, detection of the breast–chest wall boundary is a complicated problem, due to coil-related intensity inhomogeneity artefacts and partial volume issues, especially in the presence of dense breast tissue connected to the chest wall muscles and liver tissue beneath chest wall muscles [47].

The approaches published to date can be divided into three broad categories, although there is often considerable overlap.

### Methods based on image processing filters, morphological operations and geometric considerations

These techniques have the advantage that they do not require prior information concerning breast anatomy.

Twellmann et al. [39] reported a simple technique that consists of median filtering, grey-level histogram thresholding using Otsu’s method [32], and morphological closing. Better results are obtained when Otsu’s method is replaced with k-means clustering, as described by Ertas et al. [10]. Hayton et al. [16] used iterative morphological erosion followed by dilation and a “graph search” algorithm to detect the breast–air boundary and to find the approximate location of the chest wall. For certain patients, the algorithm generates satisfactory results, but it requires a long processing time and fails if the patient’s chest wall is not flat. Li et al. [27] used a simple three-class clustering method (air, parenchyma, fat) to obtain a basic segmentation, which was then refined using a gradient-based tracing algorithm, based on a set of seed points. Although there is little discussion, the method would likely fail if the seed points were incorrect. Koenig [22] employed histogram quantiles for grey-level thresholding and intensity gradients to detect breast tissue boundaries. This requires the exact location of the nipples and is able to segment the chest wall boundary only very roughly. Yao [47] refined this type of approach by fitting a spline curve and then using an active contour to improve the quality of the boundaries; they reported a system that was completely automatic. Giannini et al. [13] also described an automated system, but it seems that in both of these cases, the methods would fail for cases where parenchymal breast tissue lies in close proximity to the chest wall. Lu et al. [29] developed a method based on mathematical morphology and region growing to locate the breast–air boundary and an active contour model to locate the breast–chest wall boundary. The performance of the algorithm depends on appropriate selection of the field-of-view and makes several assumptions such as the locations of the axilla, midsternum and nipples. It fails for those patients with large breasts where the left and right sides are compressed together.

Wang et al. [42] demonstrated excellent segmentation for both large and very asymmetrical breasts, using a method based on Hessian “sheetness” filters. After detection of boundaries, breast segmentation was refined by employing an intensity-based region-growing algorithm. Wu et al. [46, 45] presented a completely automated method using a variety of edge filters. Recently, two additional methods have been published using variants of the “dynamic programming” approach. Jiang et al. [17] demonstrated a method using fat-suppressed T1-weighted images and polar transformation of curved sections of the breast wall, while Rosado-Toro et al. [35] require separate “water” and “fat” images, with an additional saturation band.

### Methods incorporating annotated atlases

Atlas-based segmentation methods, already in widespread use for the brain, have been applied to this problem. Gubern-Mérida et al. [15, 14] proposed an atlas that provides as a priori information the probabilities for each voxel to belong to pectoral muscle, heart, lungs, thorax and breast. After preprocessing (bias-field correction, normalization and registration), the breast–chest wall boundary is determined by segmenting these body structures using an atlas-based voxel classification algorithm [15]. Anatomical variations on the breast–chest wall area are captured by the atlas, but to segment the breast–air boundary, additional processing with a region-growing algorithm is employed, together with a morphological dilation filter that works slice by slice. Although the method performs well, there is a very high “startup cost” in terms of the skilled input required to set up the atlas. As the authors point out, “each volume takes approximately 45 min in a dedicated breast MRI annotation environment.”

Gallego-Ortiz and Martel [11] demonstrated an alternative atlas-based approach, incorporating entropy-based groupwise registration, maximal phase-congruency and Laplacian mapping. They applied their work to a large image cohort scanned with a Dixon MR imaging sequence. Although the computation for the results was short, the authors noted that errors were relatively high for locating the pectoral muscle boundary and there were some failed cases. The methods were refined by Khalvati et al. [18] and included the ability to obtain segmentations using two different forms of MR contrast (T1w and Dixon).

### Methods based on fuzzy c-means and machine learning

Nie et al. [30] proposed an initial segmentation based on body landmarks, followed by simple fuzzy c-means clustering [7], which incorporates a correction for intensity inhomogeneities. B-spline fitting is used to locate the chest wall; and dynamic searching removes the breast skin edge. The algorithm was well tested, but there are limitations. Appropriate selection of the field-of-view, and user interaction to determine the position of the spine, are needed. A potential issue for some breast coils is that the locations of the spinous process of the thoracic spine or the lateral margin of the bilateral pectoral muscles might be undetectable because of low signal intensity or coronal image acquisition. The authors also note that manual correction is required when contrast between the breast and chest wall is insufficient. Some of these disadvantages were mitigated in the work of Lin et al. [28]. Their method still requires input from a radiologist to identify manually the upper and lower slices containing breast tissue and manual selection of three landmarks, but the fuzzy clustering algorithm is supplemented by the creation of a thorax model to better estimate the shape of the breast–chest wall boundary. The disadvantage here is that a much larger field-of-view must be used, thus reducing the available resolution for imaging the breasts themselves.

Other literature variants of the fuzzy c-means are more difficult to assess. Klifa et al. [21, 20] provide relatively little information about the exact algorithm used, and their segmentation method is not fully automatic. Several authors combine fuzzy classification with other machine learning methods, such as support vector machines [36, 43, 41], but none of these publications presents a convincing demonstration of the segmentation of the breast from the chest wall.

Ertas et al. [9] proposed a four-element cascaded cellular neural network that performs grey-level thresholding, detection of the largest region and morphological erosion followed by reconstruction using morphological dilation.

### Novel features of this work

A number of important problems need to be addressed by any breast segmentation algorithm, including noise, partial volume effects, and bias fields related to the breast RF coil. Scan-time constraints may lead to higher levels of background noise than desirable, and this argues against any segmentation method relying purely on thresholding. Partial volume artefacts lead to ambiguities in structural definitions in the data, blurring intensities across boundaries where different tissues contribute to a single voxel or where breast tissue accounts for only a fraction of the voxel, with the rest being air. This requires careful consideration by the final consumer of the segmentation results: Is binary segmentation an appropriate output? Bias-field artefacts show up as anatomically irrelevant intensity variations in the image mainly induced by the physical properties of the receiver and transmitter coils or by variations in the local magnetic field. Since these effects are due to complex electromagnetic interactions between the imaged tissue and the acquisition system, they cannot be reduced by simple calibrations before scanning.

All of the methods mentioned above succeed to different extents in tackling these issues and are able to generate satisfactory results for certain patients. A common feature of the majority of these publications is that they describe work performed using a single MR scanner, or a small number, with none being used in the context of a large multi-centre trial.

In this paper, we describe our implementation of bias-corrected fuzzy c-means clustering (BCFCM), which minimizes the impact of the artefacts described above. The novel features of this work compared with what has gone before are as follows:

- Continuity in the selected breast region is encouraged by new additions to previous BCFCM algorithms as used in [30, 28]: (a) the BCFCM algorithm presented includes a regulariser function, operating on a *3D regulariser window*; (b) after the BCFCM classification, we add a number of morphological steps to refine the segmented volume.
- When using fuzzy c-means clustering, selection of initial class centroids is important and can have a big impact on the results. In our study, we explain a very easy but beneficial way to do this.
- We investigate the generalizability of our breast segmentation on images acquired using different brands of MR scanners in a number of clinics. In the context of multi-centre clinical trials, it is paramount that any methodology developed should be applicable to data acquired from a variety of sources, be robust against unavoidable differences in acquisition protocols and setup practice in different scanning centres, as well as catering for patients with a variety of different shapes and sizes of breast. These aspects have not previously been well studied.
- This algorithm is tested on a much larger patient cohort than for previous BCFCM methods and the statistics presented are more comprehensive.
- We demonstrate the consistency of results, using a training and test cohort.

## Materials and methods

### MR imaging protocol and patient cohort

At our institution, we have set up a case database that contains images from approximately 500 women who were scanned in the UK multi-centre study of MRI screening for breast cancer (MARIBS) [26]. T1-weighted images were acquired in the coronal plane using a predetermined 3D SPGR pulse sequence with flip angle = 35°, matrix size = 256 × 256, slice thickness = 2.5 mm and FOV = 340 mm, with typically around 85 slices per volume in the analysed breast region. Subjects were positioned prone with the breast imaged in gentle compression within dedicated breast coils [5]. All of the study participants gave consent to their anonymized images being used for research purposes. Sixteen-bit greyscale image sets were transferred from the MR scanner in DICOM format for further analysis.

Eighty-two women from the MARIBS cohort, selected to provide a range of breast fat content, were entered into this study. As summarized in Table 1, the cohort included 29 fatty, 30 fibroglandular (or “heterogeneously dense”) and 23 dense breasts in a range of sizes. T1-weighted breast MR images without fat suppression were acquired, and this subset contained data from six different models of 1.5 and 1.0 T scanners from three different manufactures (GE Medical Systems, Slough, UK; Philips Medical Systems, Reigate, UK; Siemens Medical Solutions, Bracknell, UK) and situated in 15 clinics. From these data, a training dataset, composed of 20 fatty, 16 heterogeneously dense and 14 dense breasts was used; the remaining cases formed the test dataset.

**Table 1:** Datasets categorized by breast type and MRI system manufacturer

	Siemens	Philips	GE	All
Breast type				
Fatty	14	6	9	29
Fibroglandular	14	5	11	30
Dense	11	5	7	23
All	39	16	27	82

### Fuzzy c-means algorithm for breast MR image segmentation

We chose to examine the fuzzy c-means (FCM) algorithm because of its potential in overcoming partial volume effects (something that is particularly important in later stages of the breast density calculation, after the initial whole-breast segmentation described here). FCM is one of a class of algorithms based on membership functions, which can be used for “soft” segmentations of overlapped tissue classes [34]. Among these approaches, FCM has the advantage that it can be modified to carry out a simultaneous intensity inhomogeneity compensation (bias correction) that is computationally less expensive than any prefiltering operation [33].

In the literature, FCM-based techniques with intensity inhomogeneity minimization have been primarily developed for segmentation of brain MR images. Pham and Prince [33] introduced an adaptive FCM clustering that incorporates regularization terms in the objective function. It is sensitive to noise and converges slowly. Ahmed et al. [1] developed BCFCM clustering that employs a neighbourhood regularizer in the objective function to allow labelling of a voxel to be influenced by the labels in its immediate neighbourhood in 2D. It converges faster and is insensitive to salt-and-pepper noise. BCFCM clustering assumes that the observed image intensity is a product of the intrinsic tissue signal and a spatially varying coil response factor, thus representing intensity variation artefacts as an additive bias field in the logarithmic domain. In this study, we extend the conventional 2D BCFCM to include a 3D regulariser and also include the new step of setting up the clustering parameters to generalize breast segmentation on images acquired using different MR scanners in different clinics.

Although the BCFCM algorithm does not itself require a formal training dataset, we used such an additional set (consisting of 50 cases; see Table 1) in order to gain experience on the effect of altering the different parameters and to implement refinements to the segmentation, such as post-BCFCM morphological operations. Given the large number of adjustable parameters below (*p*, *α*, ${\mathbb{W}}\left( {\text{r}} \right)$, *R*, *ε*, initial values of *β*(**r**) and class centroid offsets, together with the morphological structuring element) and the a priori unknown extent of the search space, no formal optimization procedure was employed for this work.

Consider a 3D *L* × *M* × *N* MR image and a voxel located at position **r** = (*i, j, k*), *i* = 1, 2, … ,*L*, *j* = 1, 2, … ,*M* and *k* = 1, 2, … , *N*. Let y(**r**) be the logarithm of the observed image intensity at location **r** and let *β*(**r**) be the bias field at location **r**, which is initially unknown and obtained as part of the optimization procedure. The objective function of BCFCM for 3D is given by a summation over voxels (index *r*) and fuzzy classes (index *f*):

1 $$J_{\text{BCFCM}} = \sum\limits_{r = 1}^{L \times M \times N} {\sum\limits_{f = 1}^{F} {u_{f}^{{}} ({\mathbf{r}})^{p} \left[ {D_{f} ({\mathbf{r}}) + \gamma_{f} ({\mathbf{r}})} \right]} }$$

Note that **r** = **r**(*r*), and similarly **s** = **s**(*s*) below, but these are suppressed for the purposes of clarity. Here, the first term accounts for the bias correction, while the second term is a regularizer encouraging smoothness and the cost functions are defined as follows:

2 $$D_{f} ({\mathbf{r}}) = \left\| {y({\mathbf{r}}) - \beta ({\mathbf{r}}) - c_{f} } \right\|^{2}$$

3 $$\gamma_{f} ({\mathbf{r}}) = \frac{\alpha }{R}\sum\limits_{\begin{subarray}{l} \, s = 1 \\ {\mathbf{s}}(s) \in {\mathbf{W}}({\mathbf{r)}} \end{subarray} }^{R} {\left\| {y({\mathbf{s}}) - \beta ({\mathbf{s}}) - c_{f} } \right\|^{2} }$$

*F* is the total number of tissue classes, and *f* = 1, 2, … , *F*. *u*_*f*_ (**r**) is the membership value at voxel location **r** for class *f* such that $\sum\nolimits_{f = 1}^{F} {u_{f} ({\mathbf{r}})} = 1$ and 0 ≤ *u*_*f*_(**r**) ≤ 1. *c*_*f*_ is the centroid of class *f*. In the regularizer term, ${\mathbb{W}}\left( {\text{r}} \right)$ is the set of points in a “regularizer window” placed around voxel location **r,** and *R* is the total number of entities in ${\mathbb{W}}\left( {\text{r}} \right)$. The weighting factor α and the size of the regularizer window were chosen empirically based on experience with the controls, the effect of the regularizer biasing the solution toward piecewise-homogeneous labelling. Fuzzy index *p* determines the amount of fuzziness of the resulting classification and a high value of *p* corresponds to higher fuzziness. The norm operator ||·|| stands for the standard Euclidean distance.

The minima of *J*_BCFCM_ can be numerically computed using an iterative optimization procedure started by assigning initial values to *β*(**r**) and *c*_*f*_. The iteration is stopped when the absolute change in class centroids is under a user chosen threshold *ε*. During each step, new class centroids, bias field and membership values are calculated using

4 $$c_{f}^{*} = \frac{{\sum\nolimits_{r = 1}^{L \times M \times N} {u_{f}^{{}} ({\mathbf{r}})^{p} \left( {\left[ {y({\mathbf{r}}) - \beta ({\mathbf{r}})} \right] + \frac{\alpha }{R}\sum\nolimits_{\begin{subarray}{l} s = 1 \\ {\mathbf{s}}(s) \in {\mathbf{W}}({\mathbf{r)}} \end{subarray} }^{R} {\left[ {y({\mathbf{s}}) - \beta ({\mathbf{s}})} \right]} } \right)} }}{{(1 + \alpha )\sum\nolimits_{r = 1}^{L \times M \times N} {u_{f}^{{}} ({\mathbf{r}})^{p} } }}$$

5 $$\beta^{*} ({\mathbf{r}}) = y({\mathbf{r}}) - \frac{{\sum\nolimits_{f = 1}^{F} {u_{f}^{{}} ({\mathbf{r}})^{p} c_{f} } }}{{\sum\nolimits_{f = 1}^{F} {u_{f}^{{}} ({\mathbf{r}})^{p} } }}$$

6 $$u_{f}^{*} ({\mathbf{r}}) = \frac{1}{{\sum\nolimits_{h = 1}^{F} {\left( {\frac{{D_{f} ({\mathbf{r}}) + \gamma_{f} ({\mathbf{r}})}}{{D_{h} ({\mathbf{r}}) + \gamma_{f} ({\mathbf{r}})}}} \right)^{{\frac{1}{p - 1}}} } }}$$

In this study, non fat-suppressed T1-weighted breast MR image slices (acquired originally with the “in-plane” orientation as coronal) are used to segment breast volumes. The datasets are split into two equally sized volume images in the axial plane to localize intensity inhomogeneity artefacts, thus improving the success of the extended BCFCM clustering. The extended BCFCM clustering is applied to each image slice in these volumes with *p* = 2, initial *β*(**r**) = 0.01, α = 0.20, *R* = 7 (a 3D 6-neighbourhood window) and *ε* = 0.01. Given the presence of breast in all images, two classes are defined: breast and non-breast (i.e. background) and so *F* = 2. Initial class centroids are set to the mean intensity of the processed image slice plus two offset values determined for each MR scan. For most of the cases used in the present work, the offset values of 20 and 90 give the best results. Typically, for images with significant quantities of low-intensity parenchyma, our experience was that both low offset and high offset values should be decreased. On the other hand, when the background image intensity is high, the low offset value must be increased.

Each BCFCM clustering outputs a binary image that may include some misclassified regions outside the breast and some holes inside the breast. Refinement 1 is to remove the unwanted regions and to fill the holes. 2D hole-filling followed by a 4-neighbourhood connectivity search and object labelling is performed. The object with the biggest area is identified as the breast region. Other smaller objects are removed from the binary image. The resultant images of the slices are stored and then merged to form an approximate breast volume.

**Fig. 1:**
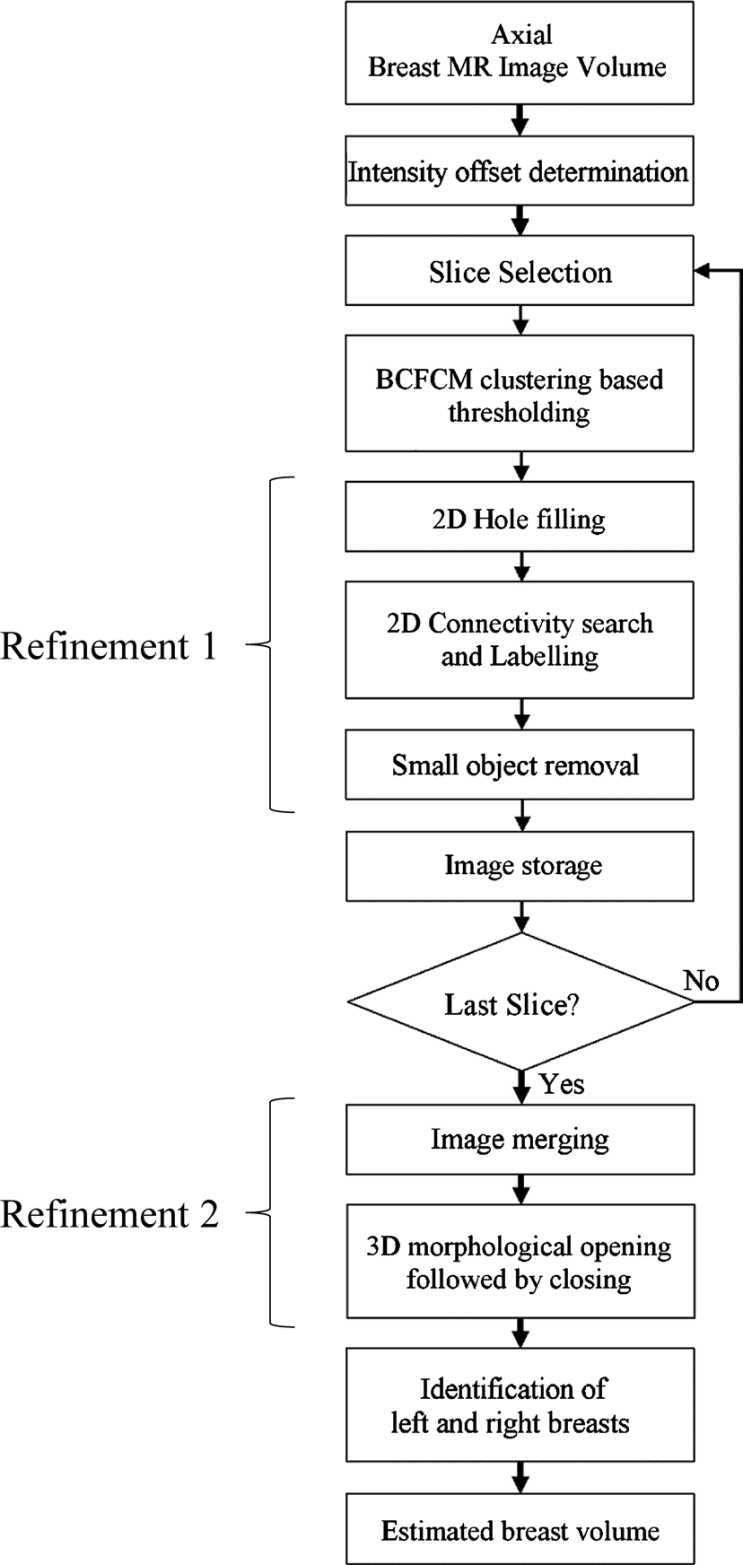
Simplified flowchart of the breast segmentation algorithm

Within the approximate breast volume, there may be some non-breast tissue segmented for cases in which breast tissue is connected to the chest wall; and there may also be some unsegmented breast tissue left for cases in which dense breast tissue is connected to the chest wall muscles. Refinement 2 is to reduce the fraction of these over- and under-segmentations, by performing 3D morphological image opening, followed by closure using two cylindrical structuring elements having the same radius of 3 voxels but different heights of 3 voxels and 25 voxels in the axial plane. (These sizes were determined after several experiments with the training dataset). The simplified flowchart showing all the steps explained above is seen in Fig. 1.

### Identification of the left and the right breast

For computer-assisted image evaluation to assess breast density or detect lesions, separate processing of the left and right breasts is also needed. These can be separated with a vertical line passing through the midsternum on the axial plane, requiring the localization of midsternum, since the use of a breast coil ensures that the sternum is roughly in the centre of the image [22, 30]. In this study, the location of the midsternum is detected automatically from the axial slice where the breast occupies the largest area in the segmentation result. First, the air–breast boundary is extracted as follows. The binary segmented volume is examined slice by slice in an axial plane. Each column in the AP direction for the given slice is inspected starting from the air side and the first transit from zero to one is recorded. These positions are plotted in the form of an air–breast boundary curve. Next, the locations of the local maxima and minima on this curve are found using the zero points of the first derivative of the curve and the sign of the second derivative. The two local maxima nearest to the curve centre are usually the nipple locations. The midsternum location is detected as the local minimum nearest to the centre of the curve and used to identify left and right breasts.

### Segmentation performance evaluation

Success of the breast segmentation is quantified with several metrics. These were computed from the region estimated automatically by the above method and the region delineated manually. In prior publications, different authors have chosen to deal with the immensity of this manual segmentation task in alternative ways. For example, in [18], “images were contoured by an expert by manual initialization of an active contour technique”, while in [15] “annotations were done every 5–10 slices and linear interpolation was applied to obtain the complete labelling”. In the study presented here, to minimize the time required for manual segmentations, manual corrections to computerized segmentations are used. These adjusted segmentations are clearly not “blind” to computer segmentations, and hence a comparison of segmentation performance with methods that use “pure” manual segmentation is not entirely fair. However, our prior experience, as reported in [9], is that, for the breast-to-air boundary, computerized segmentations and pure manual segmentations are almost the same. This method therefore allows us to process a large statistical sample, where the human reviewer performing the corrections can focus on the chest wall region, which is where automated algorithms face the greatest difficulties.

Let *C*_*s*_ be the set of voxels within the breast region estimated by our segmentation method, *C*_*r*_ be the set of voxels delineated manually and $n_{\Re }$ be the total number of voxels within region $\Re$. Relative overlap RO, as a measure of segmentation precision (also named as Jaccard similarity coefficient), is computed as in [37] using

7 $${\text{RO}} = \frac{{n_{{C_{s} \cap C_{r} }} }}{{n_{{C_{s} \cup C_{r} }} }}$$

Segmentation accuracy is assessed using the true-positive volume fraction TPVF and false-positive volume fraction FPVF calculated by [40]

8 $${\text{TPVF}} = \frac{{n_{{C_{s} \cap C_{r} }} }}{{n_{{C_{r} }} }}$$

9 $${\text{FPVF}} = \frac{{n_{{C_{s} - C_{s} \cap C_{r} }} }}{{n_{{C_{r} }} }}$$

TPVF is the fraction of the total number of voxels delineated by the expert that was included in the volume segmented by our method and FPVF is the voxels falsely identified by our method as a fraction of the amount of the voxels delineated by the expert. RO, TPVF and FPVF range from 0 to 1. However, clearly, the greater the RO and the TPVF and the smaller the FPVF values are, the better will be the segmentation.

## Results

On a personal computer with a 3 GHz Intel Pentium 4 processor and 3 GB RAM, segmentation of the whole breast of a single patient takes approximately 2 min using a numerical implementation in IDL 7.0 (ITT Visual Information Solutions, USA). The algorithm runs successfully on data from all 15 different centres included in the trial. When compared to manual segmentation that takes almost 50 min for a patient, the algorithm leads to considerable increase in the time efficiency.

**Fig. 2:**
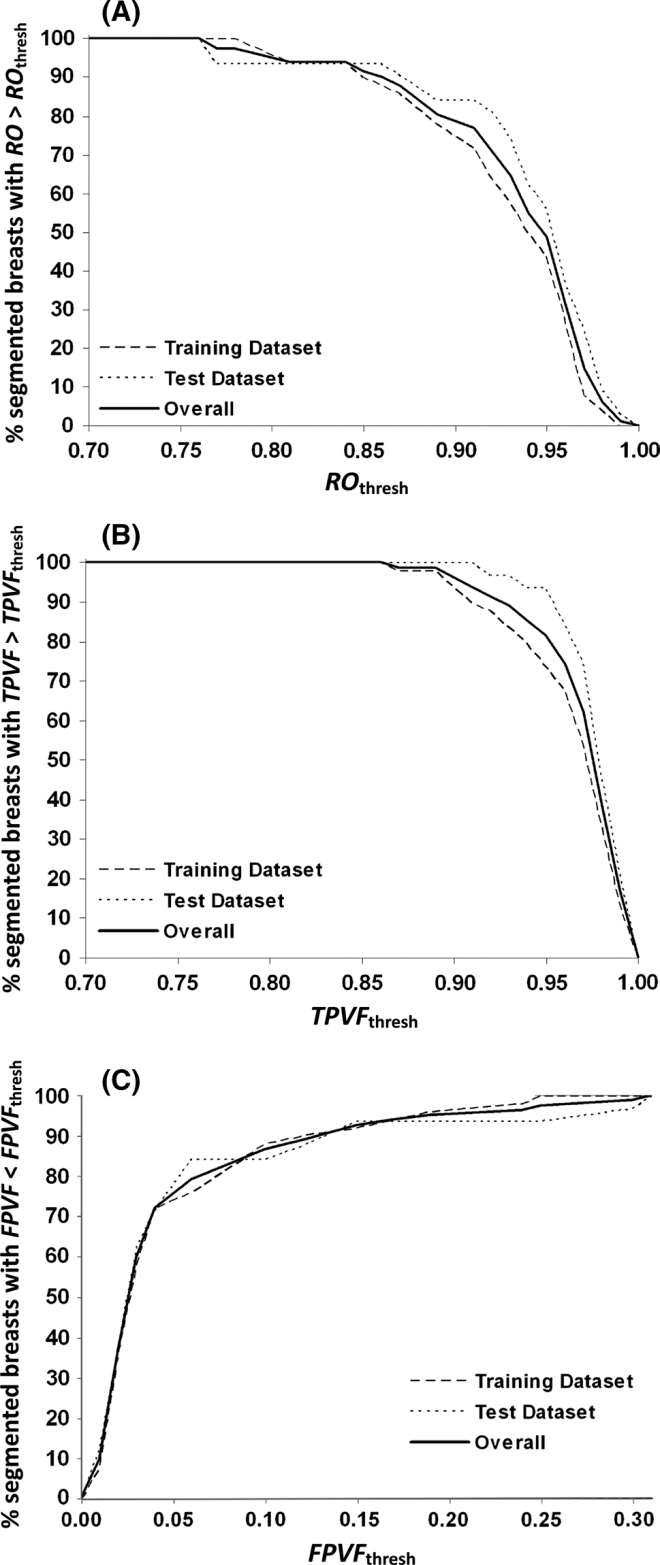
Results for: **a** relative overlap; **b** true-positive volume fraction; and **c** false-positive volume fraction

Figure 2a summarizes our results for the relative overlap (RO) statistic defined above; it provides a measure of the success of our BCFCM algorithm in matching the results of manual segmentation, as computed over the entire analysis cohort. Each position along the horizontal axis represents a threshold value RO_thresh_, and on the vertical axis, we plot the number of segmented breasts for which the calculated RO value is greater than this threshold, expressed as a percentage of the total number of breasts. Thus, for example, 100 % of the segmentations have an RO > 0.70, while only about 48 % of the breast segmentations pass at an RO threshold of 0.95.

Figure 2b, c displays corresponding results for the TPVF and FPVF statistics. Note that for a “good” algorithm, the first two graphs should remain close to 100 % for as far along the horizontal axis as possible, while for FPVF, the percentage will ideally drop to low values as early as possible.

We can also rate the overall performance of our algorithm by the mean and standard deviation of the three statistics over the whole cohort. These data are summarized in Table 2, together with the results for the different breast types.

**Table 2:** Comparison of the segmentation performance for each of the density categories of the BCFCM-based method introduced in this study and the CNN-based method previously reported in [9]

Breast type	RO_BCFCM_	TPVF_BCFCM_	FPVF_BCFCM_	RO_CNN_	TPVF_CNN_	FPVF_CNN_
Training set (*n* = 50)						
Fatty	0.95 ± 0.03	0.97 ± 0.03	0.02 ± 0.01	0.90 ± 0.06	0.92 ± 0.07	0.03 ± 0.03
Fibroglandular	0.93 ± 0.04	0.97 ± 0.03	0.04 ± 0.04	0.87 ± 0.07	0.93 ± 0.06	0.08 ± 0.08
Dense	0.90 ± 0.06	0.97 ± 0.04	0.08 ± 0.08	0.86 ± 0.05	0.93 ± 0.07	0.08 ± 0.05
All	0.93 ± 0.05	0.97 ± 0.03	0.04 ± 0.06	0.88 ± 0.06	0.93 ± 0.06	0.06 ± 0.06
Test set (*n* = 32)						
Fatty	0.97 ± 0.02	0.98 ± 0.02	0.01 ± 0.01	0.93 ± 0.04	0.94 ± 0.04	0.02 ± 0.02
Fibroglandular	0.96 ± 0.03	0.98 ± 0.01	0.03 ± 0.02	0.90 ± 0.03	0.98 ± 0.01	0.10 ± 0.05
Dense	0.90 ± 0.08	0.98 ± 0.02	0.11 ± 0.12	0.83 ± 0.11	0.92 ± 0.11	0.12 ± 0.12
All	0.94 ± 0.05	0.98 ± 0.02	0.05 ± 0.07	0.89 ± 0.07	0.95 ± 0.07	0.08 ± 0.08
Overall	0.94 ± 0.05	0.97 ± 0.03	0.04 ± 0.06	0.88 ± 0.07	0.94 ± 0.07	0.07 ± 0.07

In order to assess the degree to which the new method represents an improvement over an algorithm previously demonstrated in the literature, we performed a comparison with the cellular neural network (CNN) method [9] for all 82 cases. The adjustable parameter *b* in the thresholding stage of that algorithm was here set to 0.55, which gave significantly improved results for the current cohort, compared with the value of 0.79 in [9]. The best CNN segmentation performance gave overall statistics RO = 0.88, TPVF = 0.94 and FPVF = 0.07 on these data, which is somewhat inferior to the results of the new BCFCM method (RO = 0.94, TVPF = 0.97, FPVF = 0.04). Inspection of Table 2 demonstrates consistent improvement in performance across categories by moving to the new algorithm.

**Fig. 3:**
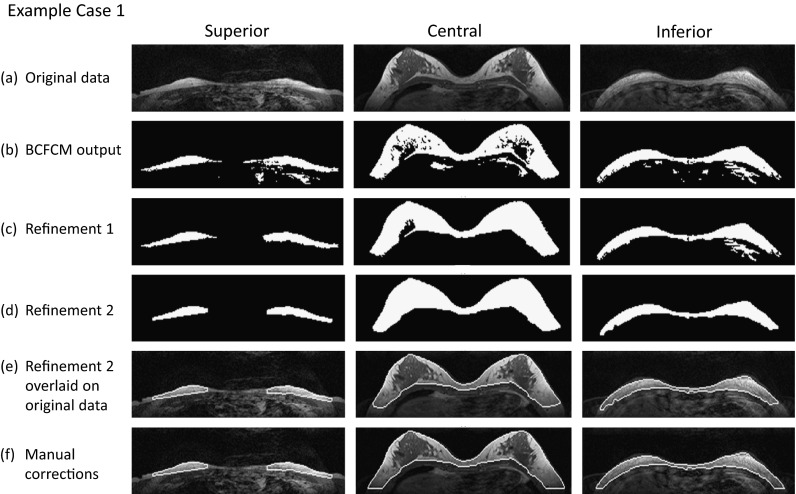
Medium-sized dense breast: **a** representative MR slices; **b** BCFCM outputs; **c** mask after Refinement 1; **d** mask after Refinement 2; **e** breast boundary from Refinement 2 superimposed onto original images; **f** manually corrected contours (RO = 0.93, TPVF = 0.94 and FPVF = 0.01)

**Fig. 4:**
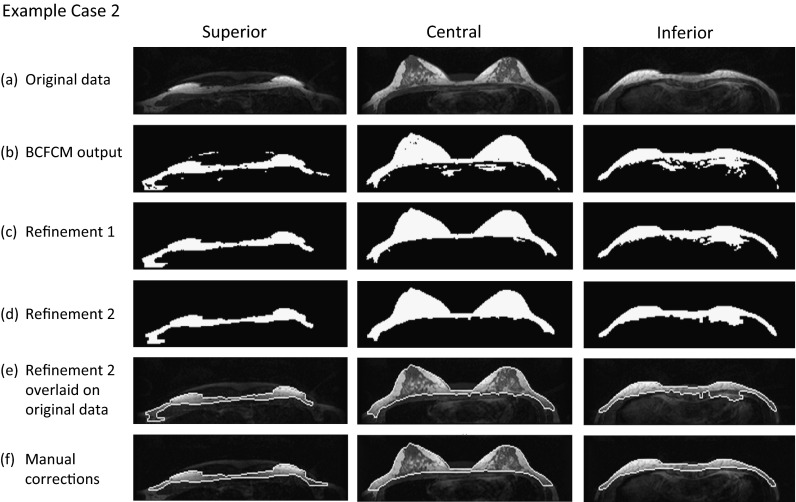
Small dense breast: rows as for Fig. 3 (RO = 0.88, TPVF = 1.00 and FPVF = 0.13)

The various stages of the segmentation algorithm developed here are illustrated in Figs. 3 and 4. Row (a) shows axial T1-weighted images from a superior slice, the middle slice in which the breast occupies the largest area, and an inferior slice; row (b) shows the initial BCFCM outputs; row (c) shows the first stage of refinement, namely 2D hole-filling followed by a 4-neighbourhood connectivity search, object labelling and small-object removal; row (d) is the final segmentation output after the second stage of refinement: 3D morphological opening; row (e) shows the bounding contours of segmentation in (d), superimposed on the original images; while (f) is the human observer’s “gold-standard” segmentation.

Our general experience is that large fatty breasts are unproblematic to segment, even when they contain “skin folds”.

The data chosen for Figs. 3 and 4 illustrate the algorithm performance in more challenging cases. We show two examples of smaller breasts with fibroglandular tissue connected to the chest wall muscle. These tend to be difficult to segment because of weak contrast boundaries. In Fig. 3, the images are noisy and corrupted by cardiac motion and partial volume artefacts. On the inferior slice, liver tissue is adjacent to the chest wall muscles. Offset values of 20 and 90 are used. Nevertheless, the automated method performs well (on average, RO = 0.93, TPVF = 0.94 and FPVF = 0.01).

By contrast, Fig. 4 shows an example of a small breast in which the automated algorithm fails to exclude completely the pectoral muscle. Fibroglandular tissue is in close proximity to the flat chest wall, and liver tissue is present right underneath the chest wall muscles. Aliasing artefacts are a significant confound. Despite these issues, using offset values 20 and 90, segmentation performance for this case is still generally good (on average, RO = 0.88, TPVF = 1.00 and FPVF = 0.13).

**Fig. 5:**
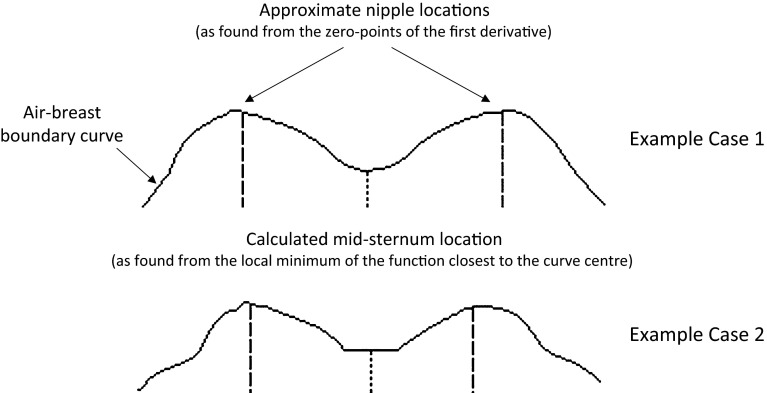
Air–breast boundary curves; computed nipples and midsternum locations (*dashed* and *dotted lines*, respectively) for the cases corresponding to (a) Fig. 3 and (b) Fig. 4

**Fig. 6:**
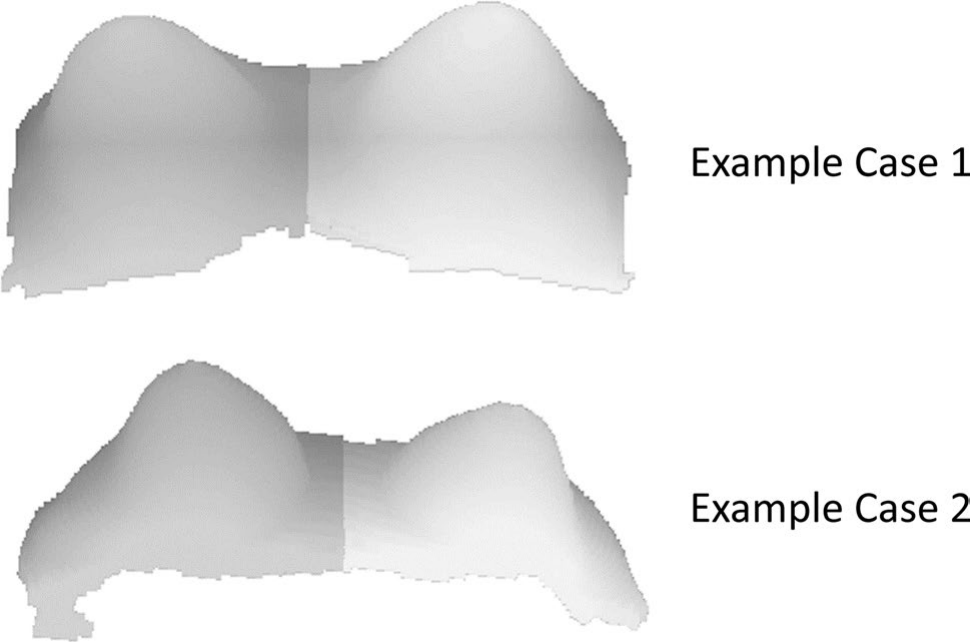
Identified *right* and *left* breasts (*grey* and *light grey* areas, respectively) for the cases corresponding to (a) Fig. 3 and (b) Fig. 4

For all cases, the air–breast boundary curve is obtained for the axial slice where the segmented breast occupies the largest area. Figure 5 shows the results of the algorithm for automatic detection of nipple and midsternum locations for the cases illustrated in Figs. 3 and 4. Volumetric views of the identified right breast (grey area) and left breast (light grey area) for the same two cases are shown in Fig. 6.

## Discussion

The use of BCFCM and morphological operations reduces the false segmentations due to the artefacts caused by noise, intensity inhomogeneity and partial volume. In combination with 2D hole-filling and largest-object detection, it also removes the breast skin edge and extensions into the liver tissue underneath the chest muscle. 3D image opening reduces the fraction of over-segmented regions and 3D image closing reduces the fraction of under-segmentations further.

From the curves in Fig. 2, objective measures of the algorithm’s performance can be obtained, permitting quality criteria for an “acceptable” segmentation to be defined on an application-specific basis. The segmentation algorithm performs well with high relative overlap, high true-positive volume fraction and low false-positive volume fraction. All datasets were segmented with RO > 0.77, and 90 % of all the datasets had an RO of 0.86 or better. The cumulative percentage curves are slightly different for the training and test datasets, so this last statistic varies from 88 % of the breasts in the “training” dataset to 94 % in the “test” dataset. The graphs in Fig. 2 give valuable insights into the distribution of failure.

Calculation of the midsternum location from the axial slice in which segmented breast occupies the largest area gives accurate estimates, since this slice is usually from the midsection of the breast, where the contrast of the chest wall muscle is strong and the signal-to-noise ratio is high. This location information can be used to define a vertical line to separate the breasts. Identified left and right breasts will provide additional information to algorithms that perform lesion localization or breast density assessment.

A number of scenarios exist in which the algorithm may lead to inaccurate segmentation. Chest wall muscles connected to the fibroglandular tissue of small and very dense breasts may result in *over-*segmentation. However, such errors would be tolerable without correction for computerized lesion detection, since vascularized tissues in the chest wall can be discriminated using morphological features such as 3D eccentricity [9].

Segmentation may end inappropriately within the axilla at an arbitrary edge for large breasts since image intensities are reduced further away from the breast coil. However, identification of the breast volume as the tissue lying between the breast–air and the breast–chest wall boundaries is *ambiguous*; there is no precise and generally accepted definition of lateral and superior/inferior extent of breast tissue, and the choice may also be application specific. For example, if comparing with digital mammography, it may be desirable to select a volume matching the region accessible to the X-ray measurement (as compressed by the mammography paddles), thus excluding the axillary tail of the breast altogether. Some authors have prescribed more specific definitions based on anatomic landmarks. For example, in [6] the coronal slice located 5 mm dorsal to the posterior margin of the midsternum could be considered as the end of the breast volume. In [30], the V-tip of spinous process of the thoracic spine, the lateral margin of the bilateral pectoral muscles and their connecting lines have been also used to define the posterior lateral margin at both sides for breast. However, there is considerable variation in body habitus which will affect the applicability of any fixed landmark system.

A second issue relating to breast-volume definition occurs at the breast–air boundary. The breast skin in this region has the potential to become thickened in the presence of benign changes [8]. On MR images, the skin may show similar signal intensity to that of fibroglandular tissue and, if not removed, is classified as “dense” tissue while assessing breast density [31]. This leads to undesirable impact on the assessments (i.e. overestimation of the breast density). The breast skin should thus be excluded from the breast volume defined. The segmentation algorithm introduced in this study outputs breast volumes with skin excluded.

There are other issues awaiting further exploration and development. Manual selection of intensity offset values in determination of initial centroids of BCFCM suffers from inter- and intra-operator variability although two-class clustering reduces the complexity of the initial guess. To automate this task, smoothed image histograms that have a number of modes equal to the desired number of classes have been proposed [34]. However, we have found that this is not applicable to breast MR images as image histograms can have a monomodal shape. On the other hand, breast regions in the outer slices may be over-segmented due to very low SNR. In such cases, a higher weighting factor for the BCFCM would improve the segmentation accuracy, although this would also increase the processing time dramatically. Changing the weighting factor adaptively may minimize the computational burden.

It should also be noted that a general problem for the segmentation community is that the ideal situation of T1-weighted images without fat suppression is, at the current time, rarely encountered in routine clinical practice. Fat-suppressed sequences will lead to difficulties for many of the algorithms so far presented in the literature, as there will be no strong boundary between the fat of the breast and the chest wall, with both appearing hypo-intense on images. Several of the larger studies [11, 18, 12] used research data acquired with a Dixon sequence from a non-clinical cohort [4] and our own work draws its data from a UK’s MARIBS screening trial [26]. An important endpoint in both of these trials was the production of breast density data, for which accurate segmentation is important. It is likely that all such specialist screening applications would be conducted using a specially tailored protocol compatible with segmentation. However, it is worth highlighting the fact that any use of segmentation within the routine clinical context might require changes in acquisition protocol that could lengthen the standard breast examination.

## Conclusion

In breast MR imaging, advanced computer-assisted image evaluation requires accurate separation of breast tissues from other tissues and regions of the body, such as chest muscle, superficial body fat, lungs, heart and ribs, that may confound analysis for breast density assessment or lesion localization. Background noise, bias fields, motion and partial volume artefacts all contribute to the potential for incorrect segmentation. Since these effects are dependent on the tissue imaged, they cannot be removed by simple calibrations before scanning. Previously reported breast segmentation methods have been demonstrated for images acquired using a particular MR scanner and are able to generate satisfactory results for certain patients and for certain degrees of these artefacts.

In this study, we have introduced an algorithm developed for breast images acquired by a range of MR scanners in multiple centres. It is based on 3D bias-corrected fuzzy c-means clustering and morphological operations and possesses a number of novel features. The full breast extent is determined on non fat-suppressed T1-weighted images without requiring any prior information concerning breast anatomy. Left and right breasts are identified separately using automatic detection of the midsternum location. The new segmentation method is fast, and statistical analysis on a large dataset shows it performs well on multi-centre and multi-instrument data.
